# Lung ultrasound in the management of mechanical ventilation in pediatric critical care: a narrative review

**DOI:** 10.3389/fped.2025.1630918

**Published:** 2025-09-05

**Authors:** Nailu Lealina Garrido Lopes, Vivian Henriques do Amaral, Marina Simões Minozzi, Paola Guazzelli Pitta Madureira, Luisa Zagne Braz, Rogerio da Hora Passos

**Affiliations:** ^1^ Departamento Materno Infantil, Einstein Hospital Israelita, Sao Paulo, Brazil; ^2^ Nucleo de Dados e Informações Gerenciais, Einstein Hospital Israelita, Sao Paulo, Brazil; ^3^ Hospital Municipal Gilson de Cassia Marques de Carvalho, Einstein Hospital Israelita, Sao Paulo, Brazil; ^4^ Instituto do Cancer do Estado de São Paulo, Universidade de São Paulo, Sao Paulo, Brazil; ^5^ Departamento de Prática Médica, Hospital Municipal Gilson de Cassia Marques de Carvalho, Einstein Hospital Israelita, São Paulo, Brazil; ^6^ UTI Pediátrica, AC Camargo Cancer Center, Sao Paulo, Brazil; ^7^ Diretoria de Qualidade, Segurança e SCIH, Einstein Hospital Israelita, Sao Paulo, Brazil; ^8^ Departamento de Pacientes Graves, Einstein Hospital Israelita, Sao Paulo, Brazil; ^9^ DaVita Tratamento Renal, Sao Paulo, Brazil

**Keywords:** ultrasonography, lung, respiration, artificial, intensive care units, pediatric, child, weaning

## Abstract

Lung ultrasound (LUS) has become an increasingly valuable tool in the management of critically ill pediatric patients, offering dynamic, radiation-free bedside evaluation of pulmonary function. This narrative review synthesizes current evidence on the application of LUS in the context of ventilation in children and neonates. Key domains include its role in determining the indication for ventilation, guiding ventilatory adjustments, assessing positive end-expiratory pressure (PEEP) response, supporting lung recruitment maneuvers, and aiding in weaning and extubation decisions. The use of LUS in diagnosing ventilator-associated pneumonia (VAP) is also addressed, highlighting characteristic sonographic findings and their limitations. The I-VENT mnemonic is proposed as a practical framework for clinicians to integrate LUS into ventilatory management. While further research is needed to standardize protocols and validate scoring systems, current evidence supports the routine use of LUS in pediatric intensive care as a safe, accessible, and informative tool for optimizing respiratory support.

## 1 Introduction

Lung ultrasound (LUS) has become an essential bedside tool in pediatric and neonatal care due to its portability, speed, absence of ionizing radiation, and diagnostic accuracy (1). In children, the partially ossified chest and reduced subcutaneous tissue provide excellent acoustic windows for pulmonary imaging (1).

Historically, the lung parenchyma was considered inaccessible to ultrasound due to the presence of air and the bony thoracic framework, which hinders sound wave propagation. However, a better understanding of ultrasound artifacts and their correlation with lung pathology has led to the growing use of LUS in respiratory assessment (2).

LUS has demonstrated high performance in diagnosing pneumonia, ARDS, bronchiolitis, atelectasis, pleural effusion, and pneumothorax in the pediatric population (4). Probe selection varies by age and region of interest, with high-frequency linear transducers preferred in neonates and convex or microconvex probes used for deeper lesions in older children (1, 5, 6). A systematic scanning approach—covering anterior, lateral, and posterior zones—is essential for comprehensive evaluation (1, 3, 6).

Despite the growing body of literature on point-of-care lung ultrasound, there is still limited synthesis of its targeted applications in the context of invasive mechanical ventilation in children. This narrative review aims to consolidate current evidence on the use of LUS to guide ventilatory management in pediatric intensive care units (PICUs), including lung recruitment maneuvers, diagnosis of ventilator-associated complications, and optimization of ventilation parameters. By exploring the specific sonographic patterns and their clinical implications, we aim to provide practical insights for integrating LUS into routine respiratory management of critically ill pediatric patients.

## 2 Lung ultrasound in mechanical ventilation

### 2.1 Mechanical ventilation indication and LUS score

Lung ultrasound (LUS) scores have been proposed to quantify lung aeration and guide clinical decisions in both adults and children. In adults, a common scoring method evaluates 12 thoracic regions (six per lung), with scores from 0 (normal aeration) to 3 (consolidation). In neonates, six regions are typically assessed, yielding a total score from 0 to 18 (Table 1) (7–9).

**Table 1:** Comparison of adult and neonatal lung ultrasound scoring systems.

Criteria	Adult LUS score (7, 8)	Neonatal LUS score (9)
Regions	6 for each lung (upper and lower parts of the anterior, lateral, and posterior regions)	3 for each lung (upper anterior, lower anterior, and lateral)
Scores	0: normal aeration/A-line pattern (presence of lung sliding with A-lines and, occasionally, an isolated B-line)	
	1: B-pattern (three or more well-spaced B-lines)	
	2: coalescent B-lines (with possible subpleural consolidations)	
	3: tissue-like pattern (complete loss of aeration/ extended consolidations)	
**Total Score**	**0–36**	**0–18**

In neonates with acute respiratory distress syndrome (ARDS), a LUS score ≥8 is associated with the need for invasive mechanical ventilation (MV), while lower scores may support a trial of non-invasive ventilation (NIV) (10). Additionally, bilateral “white lung” patterns on nasal CPAP have been predictive of NIV failure (11).

LUS also assists in confirming tracheal intubation by detecting bilateral pleural sliding and identifying misplacement (12). In pediatric ARDS, findings include diffuse B-lines, consolidations, pleural line abnormalities, and effusion. Given disease heterogeneity, semiquantitative scoring systems offer more accuracy than isolated B-line counts (13).

It's important to interpret LUS with age-related differences in mind. Infants under 6 months may display B-lines due to incomplete alveolarization, which typically normalize over time. In these cases, the diagnosis may require other complementary tests and ultrasound changes monitored over time (14).

### 2.2 Ventilation mode and diaphragm monitoring

LUS and diaphragmatic ultrasound can assist in choosing ventilator modes and optimizing support. LUS scores inversely correlate with lung compliance, suggesting that higher scores may signal reduced aeration or atelectasis (9).

Diaphragm thickening fraction (TF), calculated as the percentage change in diaphragm thickness during inspiration, serves as a marker of respiratory effort. TF between 15%–30% has been associated with shorter ventilation duration and preserved muscle mass in adults (10, 11). In preterm infants, modes such as NIV-NAVA may enhance diaphragm function through improved synchrony, with higher diaphragmatic excursion than other NIV modalities (12, 13).

Anatomical differences in neonates—such as a flatter diaphragm with steeper costal angles—necessitate careful interpretation (14). Diaphragm dysfunction (DD), marked by reduced thickening or excursion, is influenced by ventilation duration and mode. TF <20% is often used to identify dysfunction, and diaphragm thickness may decline by ∼2% per day during MV in children (15–17).

### 2.3 PEEP titration and alveolar recruitment assessment

LUS is valuable for titrating positive end-expiratory pressure (PEEP) and evaluating recruitment responses. Patients with diffuse aeration loss on LUS typically respond better to PEEP, while focal findings increase the risk of overdistension. Trials comparing LUS-guided PEEP to ARDSnet tables show better oxygenation and compliance in the LUS groups, though caution is needed in interpretation (15–18).

Bouhemad et al. proposed a reaeration score based on ultrasound pattern changes across 12 lung zones, correlating with pressure–volume curve analysis. Most reaeration is seen in anterior/lateral zones, while posterior consolidations are less responsive due to gravity and dependent atelectasis (Table 2) (15, 19).

**Table 2:** Lung ultrasound reaeration score.

1. Ultrasound lung aeration is assessed in each of the 12 lung regions before the intervention.			
*N: 5 normal pattern (normal lung aeration)*	*B1: 5 multiple well-defined either regularly spaced 7-mm apart or irregularly spaced B lines (moderate loss of lung aeration)*	*B2: 5 multiple coalescent B lines (severe loss of lung aeration)*	*C: 5 lung consolidation*
2. Ultrasound lung aeration is assessed in each of the 12 lung regions after the intervention.			
3. Ultrasound lung reaeration score is calculated as the sum of each score (for each lung region examined) according to the scale below:			
Quantification of reaeration:	**1 point**	**3 points**	**5 points**
	B1 → N	B2 → N	C → N
	B2 → B1	C → B1	
	C → B2		
Quantification of loss of aeration:	**5 point**	**3 points**	**1 points**
	N → C	N → B2	N → B1
		B1 → C	B1 → 2
			B2 → C

Serial LUS can also guide recruitment maneuvers and prevent unnecessary interventions. However, LUS does not detect hyperinflation or deep parenchymal overdistension. Thus, findings should be interpreted in conjunction with compliance, gas exchange, and clinical data (2, 18).

Despite the lack of standardized protocols in pediatrics, LUS-guided recruitment is feasible—especially in neonates—and helps minimize radiation exposure. Further research is needed to refine scoring and clinical application.

Prone positioning (PP) represents an additional strategy to improve oxygenation and regional aeration through postural recruitment. Performing LUS in the prone position is feasible and can be used to monitor its effects. Although oxygenation improvement after PP does not correlate with a specific sonographic pattern, studies show increased LUS scores in dependent regions after approximately 3 h of PP, with associated rises in the PaO_2_/FiO_2_ ratio. However, this effect may diminish after prolonged sessions (>6 h) (20–23).

### 2.4 Patient-ventilator asynchrony

Patient–ventilator asynchrony (PVA) is a mismatch between patient effort and ventilator response. Diaphragm ultrasound can help identify PVA by correlating muscle movement with airway pressure, often more feasibly than invasive monitoring or waveform analysis (11, 24, 25).

### 2.5 Weaning and extubation prediction

LUS and diaphragm ultrasound are increasingly used to assess readiness for weaning. In adults, a LUS score <13 after spontaneous breathing trial (SBT) predicts extubation success, while >17 indicates failure (9, 26). A rise in B-lines during SBT may also signal fluid overload and weaning risk.

In neonates, LUS score ≤6 before and ≤7 after nCPAP removal predicted successful weaning (25, 26). TF <23.2% and diaphragmatic excursion <6.2 mm have been associated with weaning failure in children, though thresholds vary by age and condition (27–31).

Several factors influence LUS predictive value, including timing relative to SBT, ventilatory support settings, and zone count (6–14) used during assessment.

## 3 Diagnosis of ventilator-associated pneumonia

Ventilator-associated pneumonia (VAP) is the most common hospital-acquired infection in pediatric intensive care, contributing to significant morbidity, mortality, and prolonged mechanical ventilation. Its reported incidence varies from 3% to 32% in children and up to 20% in neonates, depending on definitions and diagnostic methods used (32, 33). While adult criteria integrate clinical, radiologic, and microbiological findings, pediatric and neonatal populations lack standardized diagnostic frameworks. In neonates, particularly, radiographic and clinical signs are often nonspecific and overlap with other conditions such as RDS or sepsis, making the diagnosis especially challenging (34–37).

This diagnostic uncertainty may result in delayed treatment, increased antibiotic use, and prolonged ventilation. In this context, lung ultrasound (LUS) has emerged as a valuable tool for early, radiation-free, and bedside diagnosis of VAP. Studies report sensitivity and specificity above 90% for pneumonia detection using LUS in children, often exceeding the performance of chest radiography (35–37).

Typical sonographic findings in VAP include subpleural consolidations, often with dynamic air bronchograms, pleural effusions, and B-lines. Although these signs support the diagnosis of pneumonia, their interpretation must consider the clinical context, as similar patterns may be seen in atelectasis, ARDS, or bronchiolitis (37, 38).

To enhance diagnostic consistency, structured scoring systems have been proposed. One of the most studied is the VPLUS score, which assigns 1 point for the presence of two or more subpleural consolidations, 2 points for at least one dynamic air bronchogram, and 1 point for purulent tracheal secretions. A total score of 2 or more indicates a high probability of VAP. Two expanded versions—VPLUS-EAgram and VPLUS-EAquant—add microbiological data such as Gram stain or quantitative cultures, increasing diagnostic specificity to above 95% when scores reach 3 (38). For neonates, a Multiparametric Score was developed to integrate clinical, ultrasound, and microbiological criteria. On day 1, the score includes clinical signs such as temperature instability, changes in secretions, and respiratory deterioration (1 point each), along with ultrasound findings: subpleural consolidations over 0.5 cm, dynamic air bronchograms, and pleural effusion (2 points each). On day 3, a positive bacterial culture from tracheal aspirate adds 1 additional point. A total score above 4 on day 1 or above 5 on day 3 has shown excellent diagnostic performance (AUC >0.9) (37).

Despite its advantages, LUS is not without limitations. Findings such as consolidations and B-lines are not exclusive to pneumonia and may occur in atelectasis, ARDS, and bronchiolitis (37). Additionally, subcutaneous emphysema—a recurrent condition in Pediatric Intensive Care Units—may also compromise the quality of the ultrasound window in critically ill patients.

Moreover, LUS only detects lesions that reach the pleural surface and lie within intercostal windows, limiting its ability to evaluate apical, central, or subdiaphragmatic consolidations. In adults, this may result in up to 8% of lesions being missed (38); in children, this limitation is less pronounced but still relevant (37). Operator experience also influences diagnostic performance, especially when distinguishing subtle findings.

Nevertheless, compared with chest radiography, LUS demonstrates superior sensitivity and specificity for detecting consolidations and effusions, and allows serial monitoring of disease progression and complications (37, 38). It also enables dynamic evaluation of treatment response, guiding drainage of effusions, and identifying complications such as empyema. The integration of color Doppler can help differentiate inflammatory consolidations, which typically show preserved perfusion, from atelectasis, where vascular flow is often reduced or absent (37).

In summary, LUS is a powerful diagnostic tool for ventilator-associated pneumonia in pediatric and neonatal intensive care. Through the recognition of key findings and use of structured diagnostic scores, it enhances bedside clinical decision-making and reduces dependence on radiation-based imaging in this vulnerable population.

## 4 Practical summary: the I-VENT mnemonic

To facilitate the structured application of lung ultrasound in the management of mechanically ventilated pediatric patients, we propose the I-VENT mnemonic, which encompasses the key domains covered in this review:

I—Indication for ventilation: LUS scores can aid in identifying the need for mechanical ventilation, particularly in neonates with ARDS or those at risk of NIV failure.
V—Ventilation adjustment: LUS supports titration of ventilatory parameters, including detection of overdistension and atelectasis, as well as optimization of modes such as SIMV or NIV-NAVA.
E—Effusion and edema detection: LUS enables bedside detection of pleural effusions and interstitial syndromes, guiding fluid management and differential diagnoses.
N—Non-aerated areas: Consolidations, atelectatic regions, and regional heterogeneity can be identified, supporting decisions on positioning, recruitment, and antibiotic therapy.
T—Thoracic sliding and tube position: Visualization of pleural sliding confirms correct tracheal intubation, rules out selective intubation, and helps exclude pneumothorax.

This framework may serve as a practical bedside checklist and an educational tool to reinforce the comprehensive use of LUS throughout the continuum of ventilatory support.

## 5 Discussion

This narrative review highlights the growing and multifaceted role of lung ultrasound (LUS) in the management of mechanically ventilated pediatric patients. From guiding the indication for ventilation to optimizing PEEP, assessing recruitment, predicting weaning success, and diagnosing complications such as ventilator-associated pneumonia, LUS offers a dynamic, radiation-free, bedside tool that enhances clinical decision-making.

The pediatric and neonatal populations, with their unique anatomical characteristics and vulnerability to radiation, particularly benefit from the incorporation of LUS into routine care. Despite the promising results, certain limitations must be acknowledged, including reduced sensitivity for consolidations not adjacent to the pleura and challenges in standardizing protocols across age groups and clinical scenarios. Moreover, the absence of universally accepted pediatric-specific diagnostic criteria for conditions such as VAP underscores the importance of combining LUS findings with clinical judgment.

Future research should aim to validate existing scoring systems, establish evidence-based protocols, and integrate LUS training into pediatric critical care curricula. With ongoing advancements in portable ultrasound technology and increasing clinician familiarity, LUS is poised to become a cornerstone of respiratory management in pediatric intensive care units worldwide.

## References

[1] Bobillo-Perez S Girona-Alarcon M Rodriguez-Fanjul J Jordan I Balaguer Gargallo M. Lung ultrasound in children: what does it give us? Paediatr Respir Rev. (2020) 36:136–41. 10.1016/j.prrv.2019.09.006 31679983

[2] Dietrich CF Buda N Ciuca IM Dong Y Fang C Feldkamp A Lung ultrasound in children, WFUMB review paper (part 2). Med Ultrason. (2021) 23(4):443–52. 10.11152/mu-3059 33657190

[3] Lichtenstein DA Mezière GA. Relevance of lung ultrasound in the diagnosis of acute respiratory failure: the BLUE protocol. Chest. (2008) 134(1):117–25. 10.1378/chest.07-2800 18403664 PMC3734893

[4] Shi C Xu X Xu Y. Systematic review and meta-analysis of the accuracy of lung ultrasound and chest radiography in diagnosing community acquired pneumonia in children. Pediatr Pulmonol. (2024) 59(12):3130–47. 10.1002/ppul.27221 39239917 PMC11601018

[5] Musolino AM Tomà P De Rose C Pitaro E Boccuzzi E De Santis R Ten years of pediatric lung ultrasound: a narrative review. Front Physiol. (2022) 12:721951. 10.3389/fphys.2021.721951 35069230 PMC8770918

[6] Chidini G Raimondi F. Lung ultrasound for the sick child: less harm and more information than a radiograph. Eur J Pediatr. (2024) 183:1079–89. 10.1007/s00431-023-05377-3 38127086

[7] De Martino L Yousef N Ben-Ammar R Raimondi F Shankar-Aguilera S De Luca D. Lung ultrasound score predicts surfactant need in extremely preterm neonates. Pediatrics. (2018) 142(3):e20180463. 10.1542/peds.2018-0463 30108142

[8] Brat R Yousef N Klifa R Reynaud S Shankar-Aguilera S De Luca D. Lung ultrasonography score to evaluate oxygenation and surfactant need in neonates treated with continuous positive airway pressure. JAMA Pediatr. (2015) 169(8):e151797. 10.1001/jamapediatrics.2015.1797 26237465

[9] Soummer A Perbet S Brisson H Arbelot C Constantin JM Lu Q Ultrasound assessment of lung aeration loss during a successful weaning trial predicts postextubation distress. Crit Care Med. (2012) 40(7):2064–72. 10.1097/CCM.0b013e31824e68ae 22584759

[10] Corsini I Parri N Gozzini E Coviello C Leonardi V Poggi C Lung ultrasound for the differential diagnosis of respiratory distress in neonates. Neonatology. (2019) 115(1):77–84. 10.1159/000493001 30304736

[11] Ferrari G De Filippi G Elia F Panero F Volpicelli G Aprà F. Diaphragm ultrasound as a new index of discontinuation from mechanical ventilation. Crit Ultrasound J. (2014) 6(1):8. 10.1186/2036-7902-6-8 24949192 PMC4057909

[12] Raimondi F Migliaro F Corsini I Meneghin F Dolce P Pierri L Lung ultrasound score progress in neonatal respiratory distress syndrome. Pediatrics. (2021) 147(4):e2020030528. 10.1542/peds.2020-030528 33688032

[13] Yan C Hui R Lijuan Z Zhou Y. Lung ultrasound vs. chest X-ray in children with suspected pneumonia confirmed by chest computed tomography: a retrospective cohort study. Exp Ther Med. (2020) 19(2):1363–9. 10.3892/etm.2019.8333 32010310 PMC6966229

[14] Raimondi F Migliaro F Corsini I Meneghin F Pierri L Salomè S Neonatal lung ultrasound and surfactant administration: a pragmatic, multicenter study. Chest. (2021) 160(5):S0012-3692(21)01354–4. 10.1016/j.chest.2021.06.076 34293317

[15] Bouhemad B Brisson H Le-Guen M Arbelot C Lu Q Rouby JJ. Bedside ultrasound assessment of positive end-expiratory pressure-induced lung recruitment. Am J Respir Crit Care Med. (2011) 183(3):341–7. 10.1164/rccm.201003-0369OC 20851923

[16] York J Nugent K. Using lung ultrasound to guide PEEP determination in mechanically ventilated patients with acute respiratory distress syndrome. Southwest Respir Crit Care Chron. (2023) 11(47):10–20. 10.12746/swrccc.v11i47.1167

[17] Goligher EC Fan E Herridge MS Murray A Vorona S Brace D Evolution of diaphragm thickness during mechanical ventilation: impact of inspiratory effort. Am J Respir Crit Care Med. (2015) 192(9):1080–8. 10.1164/rccm.201503-0620OC 26167730

[18] Zimatore C Algera AG Botta M Pierrakos C Serpa Neto A Grasso S Lung ultrasound to determine the effect of lower vs. higher PEEP on lung aeration in patients without ARDS – a substudy of a randomized clinical trial. Diagnostics (Basel). (2023) 13(12):1989. 10.3390/diagnostics13121989 37370885 PMC10297592

[19] Bouhemad B Liu ZH Arbelot C Zhang M Ferarri F Le-Guen M Ultrasound assessment of antibiotic-induced pulmonary reaeration in ventilator-associated pneumonia. Crit Care Med. (2010) 38(1):84–92. 10.1097/CCM.0b013e3181b08cdb 19633538

[20] Plantinga C Klompmaker P Haaksma ME Mousa A Blok SG Heldeweg MLA Use of lung ultrasound in the new definitions of acute respiratory distress syndrome increases the occurrence rate of acute respiratory distress syndrome. Crit Care Med. (2024) 52(2):e100–4. 10.1097/CCM.0000000000006118 37962157 PMC10793806

[21] Berry L Rehnberg L Groves P Knight M Stewart M Dushianthan A. Lung ultrasound in critical care: a narrative review. Diagnostics (Basel). (2025) 15(6):755. 10.3390/diagnostics15060755 40150097 PMC11941729

[22] Ding W Shen Y Yang J He X Zhang M. Diagnosis of pneumothorax by radiography and ultrasonography: a meta-analysis. Chest. (2011) 140(4):859–66. 10.1378/chest.10-2946 21546439

[23] Mongodi S Pozzi M Orlando A Bouhemad B Stella A Tavazzi G Lung ultrasound for daily monitoring of ARDS patients on extracorporeal membrane oxygenation: preliminary experience. Intensive Care Med. (2018) 44(1):123–4. 10.1007/s00134-017-4941-7 28936711

[24] Constantin JM Grasso S Chanques G Aufort S Futier E Sebbane M Lung morphology predicts response to recruitment maneuver in patients with acute respiratory distress syndrome. Crit Care Med. (2010) 38(4):1108–17. 10.1097/CCM.0b013e3181d451ec 20154600

[25] Constantin JM Jabaudon M Lefrant JY Jaber S Quenot JP Langeron O Personalised mechanical ventilation tailored to lung morphology versus low positive end-expiratory pressure for patients with acute respiratory distress syndrome in France (the LIVE study): a multicentre, single-blind, randomised controlled trial. Lancet Respir Med. (2019) 7(10):870–80. 10.1016/S2213-2600(19)30138-9 31399381

[26] Smith MJ Hayward SA Innes SM Miller ASC. Point-of-care lung ultrasound in patients with COVID-19 – a narrative review. Anaesthesia. (2020) 75(8):1096–104. 10.1111/anae.15082 32275766 PMC7262296

[27] Volpicelli G Lamorte A Villén T. What's new in lung ultrasound during the COVID-19 pandemic. Intensive Care Med. (2020) 46(7):1445–8. 10.1007/s00134-020-06048-9 32367169 PMC7196717

[28] Singh Y Tissot C Fraga MV Yousef N Cortes RG Lopez J International evidence-based guidelines on point of care ultrasound (POCUS) for critically ill neonates and children issued by the POCUS Working Group of the European Society of Paediatric and Neonatal Intensive Care (ESPNIC). Crit Care. (2020) 24(1):65. 10.1186/s13054-020-2787-9 32093763 PMC7041196

[29] Sansone F Attanasi M Di Filippo P Sferrazza Papa GF Di Pillo S Chiarelli F. Usefulness of lung ultrasound in paediatric respiratory diseases. Diagnostics (Basel). (2021) 11(10):1783. 10.3390/diagnostics11101783 34679481 PMC8534634

[30] Laveneziana P Albuquerque A Aliverti A Babb T Barreiro E Dres M ERS statement on respiratory muscle testing at rest and during exercise. Eur Respir J. (2019) 53(6):1801214. 10.1183/13993003.01214-2018 30956204

[31] Boussuges A Gole Y Blanc P. Diaphragmatic motion studied by m-mode ultrasonography: methods, reproducibility, and normal values. Chest. (2009) 135(2):391–400. 10.1378/chest.08-1541 19017880

[32] Torres A Niederman MS Chastre J Ewig S Fernandez-Vandellos P Hanberger H International ERS/ESICM/ESCMID/ALAT guidelines for the management of hospital-acquired pneumonia and ventilator-associated pneumonia: guidelines for the management of hospital-acquired pneumonia (HAP)/ventilator-associated pneumonia (VAP) of the European Respiratory Society (ERS), European Society of Intensive Care Medicine (ESICM), European Society of Clinical Microbiology and Infectious Diseases (ESCMID) and Asociación Latinoamericana del Tórax (ALAT). Eur Respir J. (2017) 50(3):1700582. 10.1183/13993003.00582-2017 28890434

[33] Tusor N De Cunto A Basma Y Klein JL Meau-Petit V. Ventilator-associated pneumonia in neonates: the role of point of care lung ultrasound. Eur J Pediatr. (2021) 180(1):137–46. 10.1007/s00431-020-03710-8 32592026 PMC7317892

[34] Uguen J Bouscaren N Pastural G Darrieux E Lopes AA Levy Y Lung ultrasound: a potential tool in the diagnosis of ventilator-associated pneumonia in pediatric intensive care units. Pediatr Pulmonol. (2024) 59(3):758–65. 10.1002/ppul.26827 38131518

[35] Naber CE Salt MD. POCUS in the PICU: a narrative review of evidence-based bedside ultrasound techniques ready for prime-time in pediatric critical care. J Intensive Care Med. (2025) 40(4):372–8. 10.1177/08850666231224391 38193214

[36] Bhalla D Naranje P Jana M Bhalla AS. Pediatric lung ultrasonography: current perspectives. Pediatr Radiol. (2022) 52(10):2038–50. 10.1007/s00247-022-05412-9 35716179 PMC9205765

[37] Mongodi S Via G Girard M Rouquette I Misset B Braschi A Lung ultrasound for early diagnosis of ventilator-associated pneumonia. Chest. (2016) 149(4):969–80. 10.1016/j.chest.2015.12.012 26836896

[38] Ammirabile A Buonsenso D Di Mauro A. Lung ultrasound in pediatrics and neonatology: an update. Healthcare (Basel). (2021) 9(8):1015. 10.3390/healthcare9081015 34442152 PMC8391473

