# Supplementary material for: Lung ultrasound in the management of mechanical ventilation in pediatric critical care: a narrative review
Source: Front Pediatr. 2025 Sep 5;13:1630918. doi: 10.3389/fped.2025.1630918 (PMC12446649; doi:10.3389/fped.2025.1630918)
Supplement: Supplementary file 1 [file Table1.docx]

### ****APPENDIX 1 -** Glossary of Lung Ultrasound Findings for Clinical Application**

To support the practical application of lung ultrasound (LUS) in pediatric ventilatory management, this glossary summarizes key ultrasonographic signs referenced throughout the review. These definitions are intended to assist clinicians in recognizing and interpreting LUS patterns at the bedside [38].

- **A-lines**: Horizontal, repetitive echogenic lines equidistant from the pleural line. Indicative of normally aerated lung and the absence of interstitial or alveolar pathology in the scanned area. (Video 1)
- **B-lines**: Vertical, hyperechoic, laser-like artifacts arising from the pleural line and extending to the bottom of the screen without fading. They move with respiration and erase A-lines. B-lines represent increased lung density and are typically associated with interstitial syndrome, edema, or evolving consolidation. While up to 3 B-lines per interspace may be normal in healthy neonates, multiple confluent B-lines (white lung) suggest severe pathology. (Video 2)
  - **White lung**: A pattern of confluent B-lines filling the entire intercostal space, reflecting marked loss of aeration. Seen in severe respiratory distress, RDS, ARDS, or pneumonia. (Figure 1)
- **Subpleural consolidation**: Hypoechoic or tissue-like area adjacent to the pleura, often with an irregular, frayed deep border (shred sign). Suggests alveolar filling, commonly seen in pneumonia, atelectasis, or meconium aspiration syndrome. (Video 3)
- **Shred sign**: Irregular, jagged posterior margin of a subpleural consolidation, distinguishing it from well-defined masses. Highly suggestive of pneumonic consolidation.
- **Air bronchogram**: Hyperechoic, branching structures within a consolidation.
  - **Dynamic air bronchogram**: Moves with respiration; typical of pneumonia.
  - **Static air bronchogram**: Remains immobile; more often associated with atelectasis.
- **Pleural line abnormalities**: Irregular, thickened, or fragmented pleural line may reflect inflammation, interstitial edema, or fibrosis. Absence of pleural sliding may suggest pneumothorax or advanced consolidation.
- **Pleural effusion**: (Video 4)
  - **Simple**: Anechoic, free-flowing fluid between parietal and visceral pleura.
  - **Complex**: Contains septations, debris, or loculations, suggestive of empyema or complicated parapneumonic effusion.
- **Lung hepatization**: A consolidated lung segment with echotexture similar to liver, often seen in extensive pneumonia. May include air bronchograms.
- **Lung sliding**: Horizontal shimmering movement of the pleural line during respiration, indicating normal apposition and movement of the visceral and parietal pleura. Absence suggests pneumothorax, extensive consolidation, or mainstem intubation.
- **Seashore sign (M-mode)**: Normal lung motion pattern, with a granular (“sandy”) appearance below the pleural line representing lung movement, and linear “waves” above it from chest wall motion.
- **Barcode/Stratosphere sign (M-mode)**: Absence of lung sliding produces a uniform linear pattern above and below the pleural line. Characteristic of pneumothorax (may also be seen in conditions such as apnea, selective intubation and pleural adhesions). (Figure 2)
- **Lung point**: Transition zone between normal lung sliding and absent sliding, pathognomonic for pneumothorax.
- **Lung pulse**: Rhythmic movement of the pleural line synchronous with cardiac activity, indicating absence of ventilation but preserved pleural contact. Often seen in cases of severe atelectasis or mainstem bronchial obstruction.
