# Supplementary figures and images for: Lung ultrasound in the management of mechanical ventilation in pediatric critical care: a narrative review
Source: Front Pediatr. 2025 Sep 5;13:1630918. doi: 10.3389/fped.2025.1630918 (PMC12446649; doi:10.3389/fped.2025.1630918)

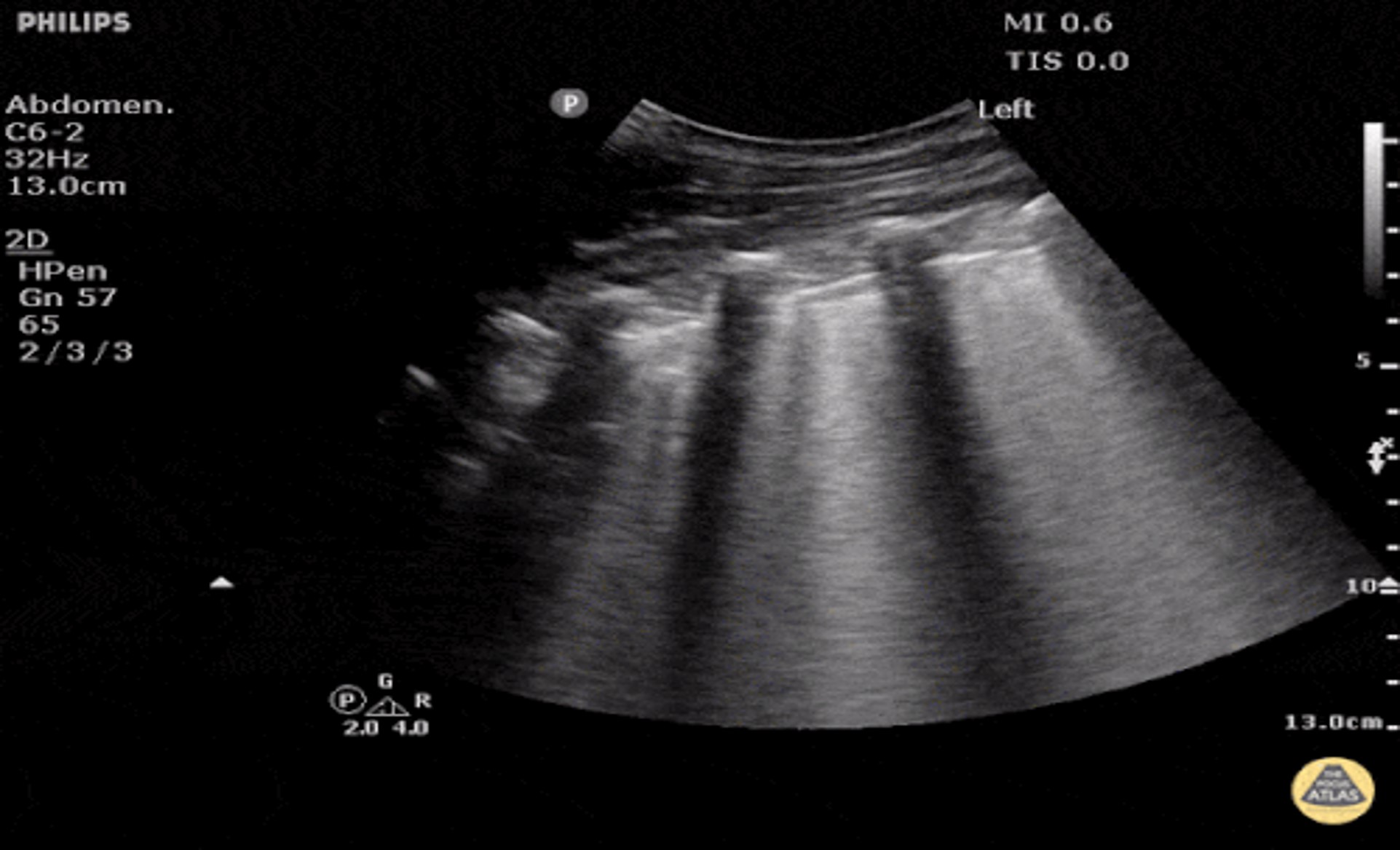

Supplement: Supplementary file 6 [file Image1.jpeg]

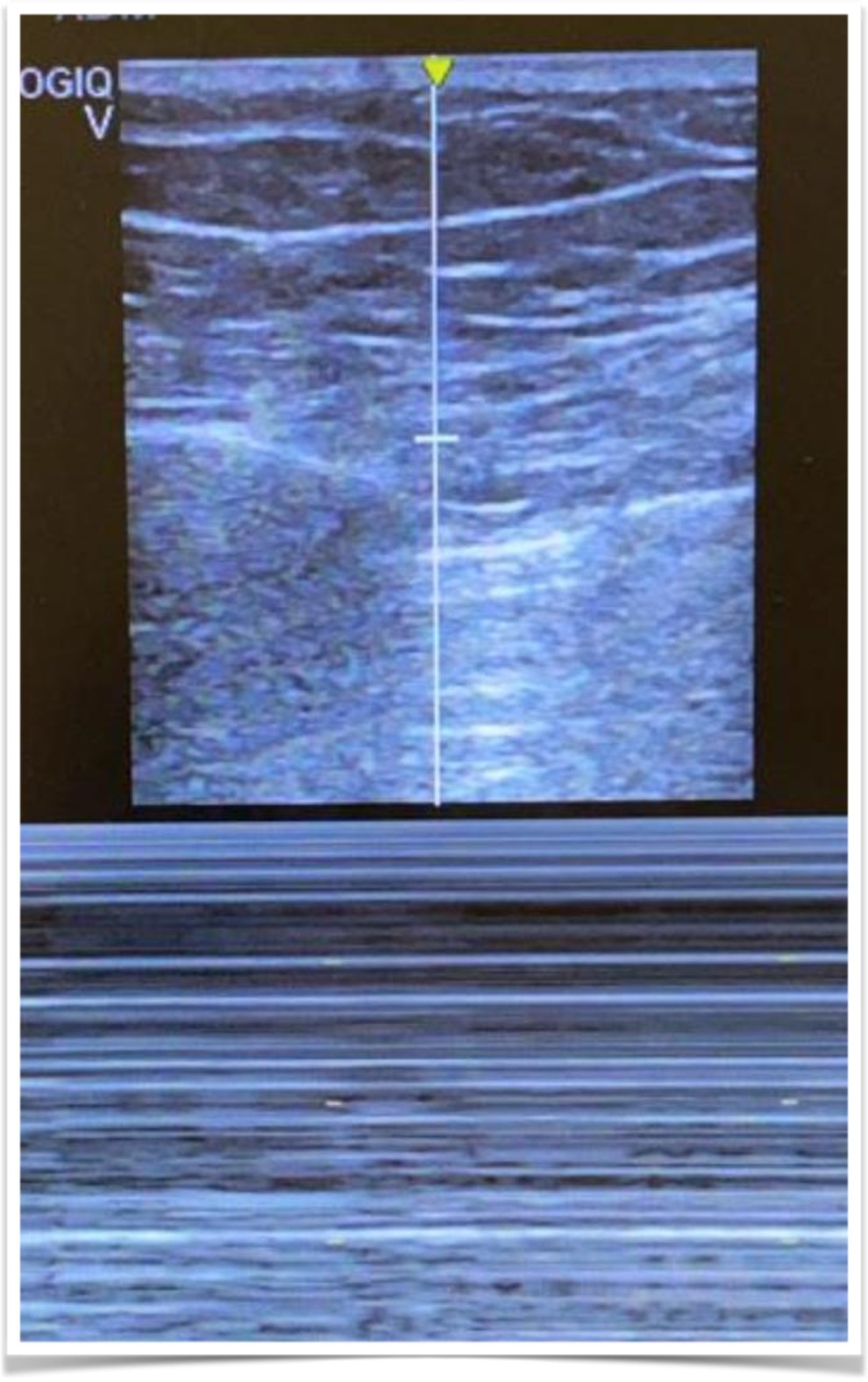

Supplement: Supplementary file 7 [file Image2.jpeg]
